# Molecular Identification and Fungal Diversity Associated with Diseases in Hass Avocado Fruit Grown in Cauca, Colombia

**DOI:** 10.3390/pathogens12121418

**Published:** 2023-12-04

**Authors:** Carolina Ángel-García, Kevin Alejandro Rodríguez-Arevalo, Nubia Murcia Riaño, Luz Natalia Martínez-Caballero, Germán Ceballos-Aguirre, Alejandro Jaramillo Laverde, Mauricio Fernando Martínez

**Affiliations:** Colombian Agricultural Research Corporation—AGROSAVIA, Palmira Research Center, Palmira 763533, Colombia; cangel@agrosavia.co (C.Á.-G.); karodriguez@agrosavia.co (K.A.R.-A.); nmurcia@agrosavia.co (N.M.R.); lnmartinez@agrosavia.co (L.N.M.-C.); gceballos@agrosavia.co (G.C.-A.); ajaramillolv@agrosavia.co (A.J.L.)

**Keywords:** *Persea americana*, diagnosis, pathogens, fungal diversity, species richness

## Abstract

Hass avocado fruit diseases are one of the main marketing constraints in Colombia. To identify and reveal the diversity of fungi associated with diseases in fruits and peduncles, symptomatic samples were collected from 67 farms in the 9 main Hass avocado-producing municipalities of the department of Cauca located in southwestern Colombia. A total of 237 monosporic isolates were obtained that were subjected to DNA extraction, amplification of the ITS region, sequencing and functional diversity analysis based on Hill numbers by municipality and altitude. The results indicated that the genera *Pseudocercospora*, *Diaporthe*, *Colletotrichum*, *Neofusiococcum*, *Lasiodiplodia* and *Pestatoliopsis* were associated with fruit diseases. The genus with the highest number of effective species was *Colletotrichum*. The ITS region revealed 100% identity of the species *Pseudocercospora norchiensis*, which was the first report of this pathogen in the crop. There was a greater richness and diversity of associated species in the three municipalities, revealing that the higher the altitude was, the lower the richness and diversity of fungi associated with fruit diseases. These results will provide a better understanding of the identification and diversity of pathogenic microorganisms present in avocado production systems in this region of Colombia.

## 1. Introduction

The avocado *Persea americana* Mill. cv. Hass has become the most important fruit of the Lauraceae family due to its organoleptic properties, high commercial value and increasing trend in fresh consumption and derivatives. Colombia is the 3rd largest grower of avocado cv. Hass in the world, with 26 producing departments, an average national yield of 10.8 ton/ha and productive potential of up to 18.8 ton/ha; exports increased 48% between 2018 and 2019, mostly destined for European markets [1,2]. However, fruit quality is the main limiting factor in the avocado trade, and the sanitary admissibility of international markets especially regulates quarantine pests and diseases or other factors that can cause postharvest losses. Fruit damage usually appears in the field and increases during harvest and postharvest, causing losses between 11.46 and 30% of the production, where lenticel damage, pest damage and peduncle and fruit diseases play an important role in the grading process [3]. The most frequent diseases that can affect avocado fruit quality are anthracnose, peca and scab, caused by the pathogens *Colletotrichum* spp., *Pseudocercospora* sp. and *Sphaceloma perseae*, respectively [2,4,5]. Furthermore, abiotic factors such as physical damage and humidity have been related to lenticel damage [6]. Several investigations have been carried out in different countries to identify pathogens associated with diseases affecting avocado fruit, among which the genera *Lasiodiplodia*, *Neofusicoccum*, *Fusarium*, *Penicillium*, *Aspergillus* and *Geotrichum* have been described [7,8,9]. In Colombia, these problems have gained special attention due to their increased incidence in the field and the rejection of fruit in processing plants, which has generated significant economic losses.

The correct identification of disease-causing agents, as well as knowledge of their distribution, is fundamental to establishing disease control and prevention methods. Identification by dichotomous keys and morphological characterization are the most widely used methods in phytopathology, based on fungal reproductive structures; however, it is a costly technique due to the plasticity in the size and shape of spores, the degree of expertise of personnel in the recognition of morphological characters and the difficulty of encouraging the sporulation of some fungi in culture media [10,11]. Currently, molecular identification is a fundamental tool in fungal taxonomy due to its universal applicability, speed and ease compared to morphology, although they are two complementary techniques for phylogeny [12]. The Consortium for the Barcode of Life established the ITS (internal transcribed spacer) region as the official code for identifying microorganisms because of its ease of amplification and sequencing and because it provides acceptable resolution in most taxa [12]. Additionally, the characterization of the functional diversity of a biological community in terms of richness and abundance can be obtained from empirical data using Hill numbers and rarefaction (interpolation) and extrapolation estimators [13], which, together with sampling curves based on size and coverage, allow for the comparison of biological communities [14].

The objective of this work was to identify fungi associated with diseases in peduncles and fruits of avocado cv. Hass grown in the department of Cauca, southwestern Colombia, using sequences of the internal transcribed spacer region (ITS) of rDNA, and to characterize the diversity through the richness and abundance of species by municipality and sampling altitude, as knowledge support for the generation of management strategies.

## 2. Materials and Methods

### 2.1. Sample Collection

Between February 2019 and August 2020, sample collection work was carried out on 67 farms in the 9 municipalities with the highest avocado production in the Cauca department located in southwest Colombia (Table S1). The crops were established on undulating land of up to 6 hectares at altitudes ranging between 1616 and 2239 m above sea level. Most farmers lacked information about the symptoms associated with diseases in peduncles and fruits and their causal agents. Despite using various strategies, such as pruning and fertilization, the main method of disease control in the study area was based on the use of synthetic chemicals to control anthracnose.

**Figure 1:**
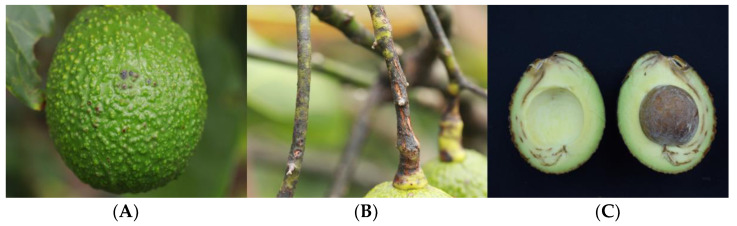
Symptoms presented in Hass avocado fruit in the field. Symptoms of lesions in lenticels (**A**), peduncle (**B**) and internal postharvest rots (**C**).

Fruit showing two types of lesions—lenticel damage and peduncle damage—were collected. Symptoms of lenticel damage were characterized by superficial light brown lesions on the lenticels, which eventually became slightly sunken and circular in shape. As these lesions grew, black, rounded, smooth and compact spots appeared on the fruit surface (Figure 1A). In the peduncles, symptoms began with the appearance of small light brown lesions, which became necrotic with slight subsidence as the infection progressed, forming a collar in the affected area (Figure 1B). Internal rots were observed after harvest and were characterized by the presence of brown to dark brown necrosis at the point of attachment to the peduncle, colonizing the vascular bundles and taking on a corky hard consistency. In some cases, there was abundant growth of cottony mycelium on the peduncle (Figure 1C).

The collection of the samples was managed under the guidelines outlined in resolution 1466 dated 3 December 2014 of the Ministry of Environment and Sustainable Development of the government of Colombia “By which a framework permit is granted for the collection of specimens of wild species of biological diversity for noncommercial scientific research purposes and other determinations are made”.

### 2.2. Isolation and Morphological Identification

All fruit and peduncle samples were disinfested by triple washing with 2% hypochlorite, 70% alcohol and sterile distilled water in a serial manner for 2 min. Two methodologies were used to isolate microorganisms from the different symptoms. (1) healthy and diseased tissue segments of approximately 2 cm were seeded in Petri dishes with potato dextrose agar (PDA; Difco^®^ 39,000 ppm), which were incubated at 24 °C for 7 days; (2) whole fruits were subjected to high humidity conditions for 15 days at 24 °C. After this time, observations were made under the Nikon SMZ745 stereoscope to identify structures of microorganisms present in the external lesions, which were seeded in Petri dishes with PDA (Difco^®^ 39,000 ppm) and incubated at 24 °C for seven days. The fruits were then cut longitudinally to evaluate the presence of internal rots in the pulp, and from these, subsamples were taken for sowing in PDA medium and incubated at 24 °C for seven days.

Once the microorganisms were grown in Petri dishes, considering their growth characteristics in culture medium, a grouping by morphotype was performed, and then monosporic cultures were obtained. Observations were made under light microscope Nikon ECLIPSE Ci-L to identify microscopic characteristics for each morphotype looking for fungal diagnostic characteristics according to Barnett and Hunter [15]. All isolates obtained were reported in the Colombian Biodiversity Information System (SiB, Colombia) [16].

### 2.3. DNA Extraction, Amplification and Sequencing of the Internal Transcribed Spacer Region (ITS) of the rDNA

Genomic DNA was obtained from 50 mg of mycelium from monosporic cultures with seven days of growth using the method described by Raeder and Broda [17] with modifications; mycelia were homogenized by grinding with a mortar and pestle in 500 μL of extraction buffer containing Tris (100 mM), EDTA (10 mM) and SDS (1%). The resulting homogenate was then transferred to a microcentrifuge tube. Subsequently, 500 μL of phenol:chloroform:isoamyl alcohol (24:24:1) was added and mixed thoroughly, and the mixture was centrifuged at 14,000 rpm for 10 min at 4 °C. The upper aqueous phase was carefully transferred to a new tube. Next, 500 μL of chloroform:isoamyl alcohol (24:1) was added, and the upper phase was again transferred to a new tube. To this, 50 μL of 3 M sodium acetate at pH 5.2 and 800 μL of cold absolute ethanol were added. The solution was gently mixed by inverting the tubes, and nucleic acids were allowed to precipitate overnight at −20 °C. The DNA was then pelleted by centrifugation at 14,000 rpm for 15 min and washed with 70% ethanol, and the pellet was air-dried at room temperature. Finally, the DNA pellet was resuspended in 30 μL of RNAse A solution (10 mg/mL), incubated at 37 °C and stored at −20 °C. The concentration and quality of DNA extracted from the 237 isolates was analyzed by electrophoresis on 1% agarose gels in Labnet^®^ chambers and by spectrophotometry with A260/280 and A260/230 absorbance ratios (Nanodrop 1000, Thermo Scientific, Waltham, MA, USA). 

For fungal identification, the ITS1-5 region was used. 8S-ITS2 (transcribed internal spacers), amplified by polymerase chain reaction (PCR) with the oligonucleotides ITS1 (3′ TCCGTAGGTGAACCTGCGG 5′) and ITS4 (5′ TCCTCCGCTTATTGATATATGC [18]; PCR amplifications were carried out in a final volume of 25 μL, for each reaction 20 ng of genomic DNA, 1X Taq buffer plus KCl, 1.5 mM MgCl_2_, 0.2 mM dNTPs, 0.2 μM of each ITS and 1 U of recombinant Taq DNA polymerase (Thermo Scientific). The PCR program consisted of initial denaturation at 94 °C for 5 min, 35 cycles at 94 °C for 45 s, 55 °C for 30 s and 72 °C for 1 min, with final extension at 72 °C for 10 min. The amplicons were validated by electrophoresis on 1.6% agarose gels, and subsequently sequenced in both directions using the Sanger method [19]. Sequences were deposited in GenBank, and accession numbers are shown in Table S2.

### 2.4. Identity and Taxonomic Positioning of Isolates

Sense and antisense DNA sequences were analyzed, edited and consolidated using Geneious Prime^®^ version 2022.2.1 software. The results were compared in the GenBank database using BLAST software version 2.15.0 available at NCBI [20]. Genus and species assignment was based on the closest e-value to zero, highest bit score, highest coverage and percent identity; sequences were then grouped into operational taxonomic units (OTU) by sensitivity analysis with alignment greater than 97% identity obtained in Geneious Prime software version 2022.0.1. Low-quality sequences were eliminated. For the construction of the taxonomic tree, alignment was performed with the Clustal W algorithm, and the nucleotide substitution model that best fit the data was determined using jModelTest software version 2.1.10 [21]. Phylogenetic reconstruction was obtained by Bayesian inference with the MrBayes program [22] by the Markov chain Monte Carlo method (MCMC). We ran 1x105 generations, sampling trees every 100 generations and extended until the deviation was less than 0.001, with a burn-in of 25%.

From the intraspecific variability of OTUs and the number of base pairs of the ITS region based on molecular identification, the dendrogram was constructed with the GraPhlAn V 1.1.3 package in Python V 3.11.6 [23].

### 2.5. Estimation of Diversity in Communities

Abundance data for each OTU were recorded by municipality and altitude category, i.e., (1613–1793, 1794–1973,1974–2153 and 2154–2333 masl). The richness and diversity index were calculated based on Hill numbers of order q = 0, q = 1 and q = 2, representing species richness, Shannon’s entropy exponential and Simpson’s diversity, respectively [13]. The analysis was performed locally with the iNEXT library [14] for R V 2022.07.0 [24], and the ggiNEXT extension was edited to plot the model prediction (extrapolation) and rarefaction (interpolation) curves for the nine municipalities. The number of common and unique OTUs in each sample was counted, and a Venn diagram was constructed.

## 3. Results

### 3.1. Isolation of Microorganisms

A total of 237 isolates associated with external and internal symptoms on fruit were obtained and classified into 12 genera (Table 1). The most frequent genera were *Colletotrichum*, which was isolated from all fruit tissues, including postharvest pulp, and *Pseudocercospora*, which was obtained mainly from lenticel lesions and initial peduncle damage. Associated with internal rots, 46 isolates of the Botryosphaeriaceae family were grouped, represented in the genera *Lasiodiplodia* and *Neofusicoccum*, with frequencies of 8.44% and 10.13%, respectively. Other genera that occurred more frequently in fruit damage were *Diaporthe* (15.61%) and *Fusarium* (13.50%). The genus *Pestalotiopsis*/*Neopestalotiopsis* occurred with a frequency of 10.97%. The saprophytes *Penicillium*, *Sarocladium* and *Schizophyllum* were found with a frequency of less than 1%.

### 3.2. Molecular Identification and Taxonomic Positioning

**Figure 2:**
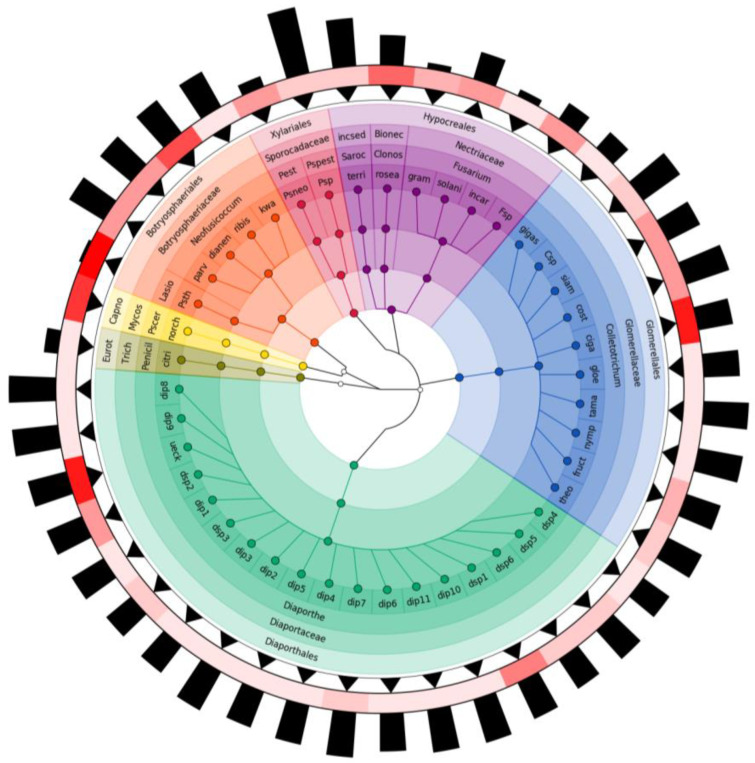
Taxonomic tree of fungi associated with diseases on peduncles and fruits of avocado cv. Hass grown in Cauca, Colombia. The colors represent the order recorded and each node the possible species according to phylogenetic analysis and BLAST comparisons. The first ring represents high (▲) or low (▼) intraspecific variability. The second ring represents the abundance of the isolates for node; the higher the abundance the greater the color intensity. The third ring represents the difference in number of nucleotides in the consensus sequences per node; the minimum size is 512pb. Abbreviations: Eurot—Eurotiales; Trich—Trichocomaceae; Penicil—*Penicillium*; citri—*citrinum*; Capno—Capnodiales; Mycos—Mycosphaerellaceae; Pscer—*Pseudocercospora*; norch—*norchiensis*; Lasio—*Lasiodiplodia*; Psth—*pseudotheobromae*; parv—*parvum*; dianen—*dinensis*; kwa—*kwambonambiense*; Pest—*Pestalotiopsis*; Psneo—*Neopestalotiopsis* sp.; Pspest—*Pseudopestalotiopsis*; Psp—*Pseudopestalotiopsis* sp.; incse—Incertae Sedis; Saroc—*Sarocladium*; terri—*terricola*; Bionec—Bionectriaceae; Clonos—*Clonostachys*; gram—*graminearum*; incar—*incarnatum*; Fsp—*Fusarium* sp.; gigas—*gigasporum*; Csp—*Colletotrichum* sp.; siam—*siamense*; cost—*costaricense*; ciga—*C. kahawae* sub. sp. *cigarro*; gloe—*gloeosporioides*; tama—*tamarilloi*; nymp—*nymphae*; fruct—*fructicola*; theo—*theobromicola*; dsp1 to 6 and dip1 to 11—*Diaporthe* spp.; ueck—*D. ueckerae*.

The DNA extraction method used resulted in obtaining high-quality DNA at an average concentration of 1345 (+948) ng/μL, with absorbance ratios within the expected ranges. Consensus sequence sensitivity analysis yielded 58 taxonomic operational units (OTUs) with 100% similarity (Table 1). Alignment of the 237 sequences using ClustalW resulted in a data set of 696 nucleotide positions with 235 conserved sites, 416 variable sites, 352 parsimony informative sites and 62 unique sites. The optimal nucleotide substitution model inferred according to the Akaike information corrected criterion (AICc) was GTR (general time reversible) with gamma distribution and invariant sites (GTR + G + I) (Figure 2).

There was greater intraspecific variability in the sequences of the genus *Diaporthe,* with a representation of 17 nodes, of which only 1 node was associated with the species *D. ueckerae*; in contrast, *Pseudocercospora* was the genus with the highest number of isolates and low intragenic variation, which revealed 100% identity with the species *P. norchiensis*. 

In the phylogenetic analysis, the genus *Colletotrichum* presented a greater number of associated species, and the sequences showed differences with respect to the number of nucleotides and were positioned in the clades of the species complexes *C. gloeosporioides*, *C. acutatum* and *C. gigasporum*, with 29, 10 and 1 isolates, respectively. At the species level, it was possible to associate *C. costarisense*, *C. fructicola*, *C. gloeosporioides* s.l., *C. kahawae* subsp. *cigarro*, *C. siamense*, *C. theobromicola* and *C. tamarilloi*.

### 3.3. Estimation of Diversity in Communities 

**Figure 3:**
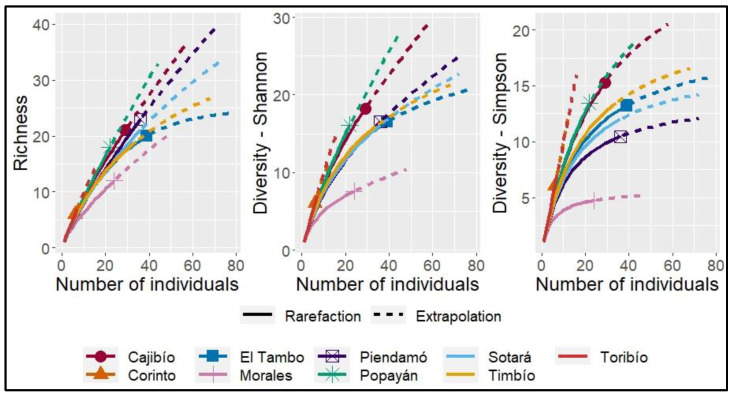
Sampling curves based on rarefaction sample size (solid line) and extrapolation (dashed line) at twice the reference sample size, of order q = 0, q = 1 and q = 2 for each of the municipalities.

The estimated sample coverage (SC. Est) for the analysis by municipality was higher than 78% for all municipalities except Corinto, for which 51% was obtained. When the analysis was performed by altitude, all clusters had an SC. Est above 80%. The highest estimated richness (q = 0) was observed in Popayán, Piendamó and Cajibío, and the lowest was observed in the municipalities of Toribío and Corinto. The diversity estimated by the exponential of Shannon’s entropy (q = 1) showed proportionality to richness, although Toribío and Cajibío were positioned as the municipalities with the lowest and highest diversity, respectively. Simpson’s inverse concentration (q = 2) placed Toribío and Morales as the least diverse municipalities, with dominance of *Diaporthe* spp. and *P. norchiensis*. On the other hand, the index positioned Cajibío as the most diverse (Table 2, Figure 3). 

**Figure 4:**
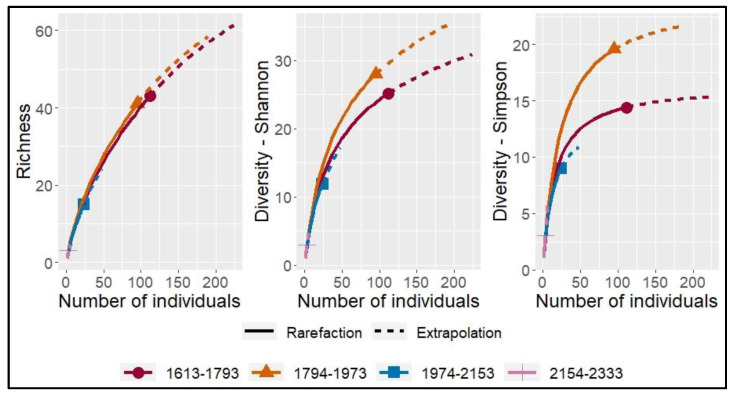
Sampling curves based on rarefaction sample size (solid line) and extrapolation (dashed line) at twice the reference sample size, of order q = 0, q = 1 and q = 2 for each of the height intervals (1613–1793), (1794–1973), (1974–2153) and (2154–2333) masl.

When the grouping of assemblages was performed by altitude intervals, their richness (q = 0), Shannon diversity (q = 1) and Simpson (q = 2) decreased as the altitude at which the sampling sites were located increased, varying from 29.53 to 4.41, 19.89 to 4.98 and 15.08 to 6.00 for q = 1, q = 2 and q = 3, respectively (Table 2, Figure 4), albeit with very small differences between them overlapping at bootstrap confidence limits. A slight increase in both richness and diversity was observed in the interval 1794–2153 masl with respect to 1613–1793 masl (Table 2, Figure 4). For all cases, the rarefaction curves for all townships and altitudes were flat and saturated, confirming that the sampling depth and sequence readings were at appropriate levels. To improve diversity estimation, further studies should consider high-throughput sequencing approaches to improve cluster resolution.

**Figure 5:**
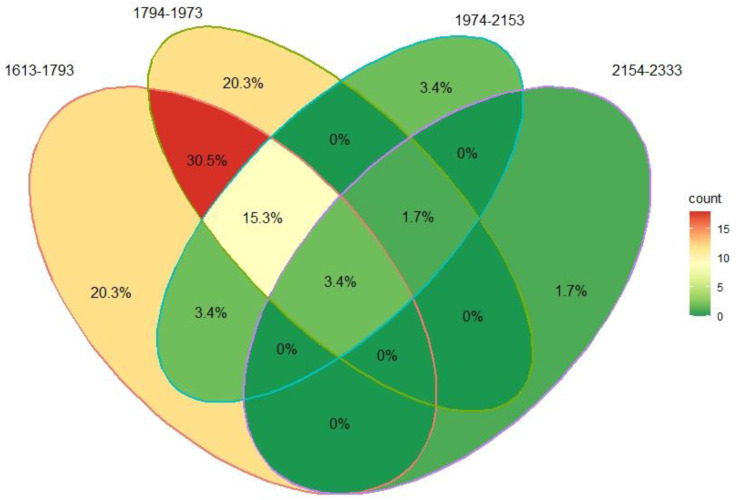
Venn diagram showing the distribution of unique and shared fungi associated with avocado fruit diseases by sampling altitude in meters above sea level.

Based on the total number of OTUs, a Venn diagram was made to represent the shared and unique effective species of the fungal groups studied (Figure 5). The results indicate that 3.4% of the fungi were common for all the municipalities studied and the four altitude ranges evaluated. The common microorganisms are *P. norchiensis*, *Diaporthe* spp. and *Fusarium* spp. In the altitudinal range between 1613 and 2153 masl, a similarity of 15.3% of the fungal species was found. When the similarity was determined in the range between 1613 and 1973 masl, 30.5% of the identified fungi were found to be shared. In this same range, the highest number of effective or unique fungal species was found, with a value of 20.3%, as shown in Figure 5.

## 4. Discussion

Genetic identification by DNA sequences represents one of the most viable techniques in taxonomy, using universal primers for amplification of genes or partial regions and sequencing. However, sequences may present a high degree of variation in response to biological and evolutionary processes that make species assignment difficult. According to the theory of Ganley and Kobayashi [25], fungal intragenomic variation in the rDNA cistron, including the tandemly repeated ITS, follows the principle of concerted evolution, and the proportion of variant ITS copies tends to be very low in most species studied. Some point mutations may be a consequence of the use of DNA polymerases that introduce stochastic errors during DNA replication, and it has been shown that the error rate of Taq polymerase varies between 0.1 and 0.01% [12]. However, in this study, the sequence heterogeneity of the ITS region in the genus *Diaporthe* was 22%, which allowed the correct separation of OTUs by interspecific but not intraspecific variation with the established cutoff values (97–99% identity); therefore, it was not possible to obtain a consensus sequence of the ITS region by Sanger sequencing for any of the OTUs identified as *Diaporthe*/*Phomopsis* spp. In contrast, *Pseudocercospora norchiensis* was the most frequent species in avocado fruit and peduncle lesions, with no intragenic variation and with 100% identity disclosure for the species. This suggests that there is low variability in the ITS region of *P. norchiensis* in the study area. *Pseudocercospora* is characterized as a genus with a limited host range by species. In avocado fruits, the causal agent of peca disease reported in Colombia, Mexico and Argentina has been *P. purpurea* [5,26], but without the disclosure of molecular identification, and *Pseudocercospora* spp. molecularly identified with primers ITS1 and ITS2 [27]. *P. norchiensis* has been reported as a pathogen in the Myrtaceae and Rosaceae [28]. This is the first molecular report of *P. norchiensis* isolated from diseases in avocado fruits cv. Hass grown in Colombia.

More than twenty species of *Colletotrichum* have been identified as causal agents of avocado anthracnose worldwide [29]. All *Colletotrichum* species identified in this study have been previously reported as causal agents, except for *C. tamarilloi*, which has been previously isolated from solanaceous fruits in Colombia [30], and the *C. gigasporum* complex, which has only been reported as a pathogen of avocado in Sri Lanka [31] and in other crops, such as coffee, tree tomato, cocoa and banana [32]. The high intraspecific variability of the ITS region does not allow taxonomic resolution to be obtained to reach the species level in this genus [33]. 

In this study, a large collection of isolates associated with the Botryosphaeriaceae family was obtained, represented by the genera *Lasiodiplodia* and *Neofusicoccum*. Species of the genus *Neofusicococcum* have also been reported to affect branches, stems, new shoots and fruits of avocado and other species, such as citrus and eucalyptus, considered the family of timber pathogens, with a wide host range and which are also difficult to differentiate morphologically due to the absence of the sexual stage [34]. In the postharvest of Hass avocado, the genus *Lasiodiplodia* and *Colletotrichum* has the highest incidence of fruit rots in the export and domestic markets in Colombia [35]. This problem is highly limiting in economic terms due to fruit rejection, which can reach up to 70.1% at the packinghouse level [3].

Species diversity in biological communities is key to understanding ecosystem processes and the impact of environmental stress or disturbance. Greater functional diversity implies greater differences among species’ trait values, distinct ecological functions and therefore potentially better functional stability in the face of disturbances caused by human impacts or environmental stresses [36]. The analysis of the species diversity of fungi associated with avocado fruit diseases represents a novel method in phytopathology to identify the dominance of genera and species, to know the distribution and genetic variations, to identify new disease-causing species, to focus on pathogenicity and virulence tests and to recommend prevention and control methods to reduce losses. To date, only the diversity of endophytic fungi of avocado plants has been explored to determine the potential of isolates as biocontrollers of *Phytophthora cinnamomi* [37] and to compare microbiomes of avocado produced by organic vs. conventional agriculture [38].

Diversity indices reveal that the dominant species represented by the Simpson index are lower in the analysis by municipality and sampling altitude, while the contribution of effective species to the spectrum is greater than the abundance proportion in the community (q = 0 > q = 2), except for the Corinto municipality and the highest sampling altitude range (2154–2333 masl), where abundance is greater than richness in the number of effective species (Figure 2 and Figure 3). Geographical location and altitudinal gradients that affect climatic conditions influence the growth and development of plant species and microorganisms and can affect interspecific competition between fungi and the occurrence of diseases, as well as the distribution and abundance of a microbial community [39]. In Colombia, a proportional relationship between fruit caliber in terms of size and volume associated with crops grown at elevations above 2300 maslhas been reported [40]. In our study, the richness and diversity of microorganisms associated with fruit and peduncle diseases has an inverse proportional relationship with the altitude of crop establishment. This study shows that Hass avocado cultivars at higher altitudes have a lower richness and diversity of fungi associated with fruit and peduncle diseases, which is of great importance to establish areas with biological aptitude for crop development.

## 5. Conclusions

The findings of this study reveal that the prevalent genera associated with diseases in Hass avocado peduncles and fruits are *Pseudocercospora* and *Colletotrichum* in equal proportion, the former with a higher species abundance and the latter with a higher number of effective species. These results indicate the potential threat they pose to the crop and should be considered in integrated disease management programs. The agreement between biogeographical and phylogenetic data allows to conclude that higher altitudes are linked to lower richness and diversity of fungi associated with avocado fruit pathologies.

## Figures and Tables

**Table 1:** Frequency of the main genera isolated from Hass avocado fruit in 67 farms in the department of Cauca, southwestern Colombia.

Associated Genus	Number of Isolations	Percentage of Population	Number of OTUs
*Colletotrichum* spp.	38	1.03	15
*Pseudocercospora* sp.	38	16.03	2
*Neofusicoccum* spp.	26	10.97	6
*Lasiodiplodia* spp.	20	8.44	3
*Diaporthe* spp.	37	15.61	17
*Pestalotiopsis* sp.	2	0.84	1
*Neopestalotiopsis* sp.	24	10.13	1
*Fusarium* spp.	32	13.50	9
*Clonostachys* sp.	16	6.75	1
*Penicillium* sp.	1	0.42	1
*Sarocladium* sp.	2	0.84	1
*Schizophyllum* sp.	1	0.42	1
∑	237	100	58

**Table 2:** Comparison of coverage estimate, richness (q0), exponential of Shannon’s entropy index (q1) and inverse of Simpson’s concentration index (q2) between municipalities and altitudes. The 95% confidence values were obtained with the bootstrap method with 1000 replications.

Assemblages	* SC	m	SC.Est	Richness (q0)	Diversity (q1)	Diversity (q2)
Diversity analysis by municipality						
Cajibío	0.75	48	0.83	17.4 ± 7.74	13.45 ± 6.07	11.33 ± 5.07
Corinto	0.11	10	0.51	8.56 ± 3.63	9.06 ± 4.09	10.00 ± 5.14
El Tambo	0.89	74	0.96	13.48 ± 4.85	9.18 ± 2.82	7.02 ± 2.44
Morales	0.72	48	0.84	16.23 ± 7.18	9.15 ± 4.87	5.04 ± 3.64
Piendamó	0.83	70	0.91	17.31 ± 6.41	11.57 ± 4.00	8.43 ± 3.39
Popayán	0.63	42	0.78	18.17 ± 7.32	13.21 ± 6.26	9.30 ± 5.37
Sotará	0.94	56	1	9.61 ± 3.36	8.28 ± 2.21	7.01 ± 2.18
Timbío	0.88	64	0.96	14.47 ± 5.03	11.96 ± 3.06	10.53 ± 3.03
Toribío	0.78	16	0.92	5.15 ± 2.99	4.16 ± 2.51	3.37 ± 2.27
Diversity by altitude intervals (masl)						
1613–1793	0.94	224	0.98	29.14 ± 6.65	18.39 ± 3.30	12.77 ± 3.27
1794–1973	0.92	190	0.98	29.53 ± 7.15	19.89 ± 3.60	15.08 ± 3.34
1974–2153	0.76	48	0.91	15.68 ± 5.80	12.41 ± 4.44	9.44 ± 4.20
2154–2333	0.33	6	0.8	4.41 ± 2.28	4.98 ± 2.74	6.00 ± 3.74

* SC—estimated sample coverage for the reference sample, m—sample size used for which the diversity order is calculated, SC.Est—estimated sample coverage for a sample size m.

## Data Availability

Data are contained within the article and Supplementary Materials.

## Supplementary Materials

The following supporting information can be downloaded at: https://www.mdpi.com/article/10.3390/pathogens12121418/s1, Table S1: Sampling location in nine municipalities in the department of Cauca, southwestern Colombia; Table S2: Fungi associated with diseases in Hass avocado fruit.
