# Supplementary material for: Molecular Identification and Fungal Diversity Associated with Diseases in Hass Avocado Fruit Grown in Cauca, Colombia
Source: Pathogens. 2023 Dec 4;12(12):1418. doi: 10.3390/pathogens12121418 (PMC10745791; doi:10.3390/pathogens12121418)
Supplement: Supplementary file 1 [file pathogens-12-01418-s001.zip › Table S1.pdf]

Table S1. Sampling location in nine municipalities in the department of Cauca, southwestern Colombia.

Subregion	Municipality	Village	Latitude	Longitude	masl*
North	Corinto	La Estér	2º 06' 22,8''	76º 14' 00,6''	1867
		La Estér	2º 06' 22,7''	76º 14' 00,5''	1867
	Toribío	Sesteadero	2º 57' 14,0''	76º 14' 33,1''	2239
		Sesteadero	2º 56' 45,7''	76º 15' 09,5''	1855
Center	Cajibío	El Lago	2° 34' 26,2''	76° 36' 16,7''	1734
		La María	2° 38' 01,2''	76° 36' 24,5''	1823
		La María	2° 38' 13,2''	76° 36' 43,6''	1808
		La María	2° 37' 57,5''	76° 36' 16,5''	1827
		La María	2° 38' 16,5''	76° 36' 05,2''	1771
		La María	2° 38' 16,2''	76° 36' 04,1''	1771
	El Tambo	Campoalegre	2º 27' 01,2''	76º 44' 19,0''	1695
		Puente Alta	2º 27' 07,2''	76º 46' 18,6''	1693
		Puente Alta	2º 27' 24,0''	76º 46' 41,0''	1716
		Las Piedras	2º 25' 55,0''	76º 44' 58,8''	1730
		Cabuyal	2° 24' 57,4''	76° 44' 35,3''	1757
		Las Piedras	2° 25' 47,3''	76° 44' 56,2''	1663
		Las Piedras	2° 25' 46,6''	76° 45' 00,0''	1718
		Las Piedras	2° 25' 56,1''	76° 45' 07,6''	1730
		Puente Alta	2° 27' 24,6''	76° 46' 35,3''	1695
		Puente Alta	2° 27' 51,9''	76° 47' 29,8''	1710
		Puente Alta	2° 26' 30,6''	76° 46´58,1''	1709
	Morales	Carpintero	2° 27' 50,0''	76° 36' 26,7''	1616
		Carpintero	2° 47' 12,3''	76° 36' 13,1''	1633
		San Antonio	2° 45' 36,3''	76° 35' 24,2''	1655
		El Rosal	2° 43' 17,4''	76° 36' 31,4''	1693
		Carpintero	2° 47' 27,4''	76° 35' 57,5''	1616
		Carpintero	2° 47' 52,4''	76° 36' 35,6''	1613
		Carpintero	2° 47' 29,0''	76° 36' 24,2''	1623
	Piendamó	la Florida	2º 39' 24,9''	76º 33' 19,3''	1836
		Octavio	2º 42' 11,0''	76º 33' 26,6''	1729
		Uvales	2º 42' 56,4''	76º 34' 03,3''	1699
		Octavio	2° 41' 50,0''	76° 33' 12,4''	1700
		San Isidro	2° 41' 10,6''	76° 34' 57,7''	1746
		Octavio	2° 42' 29,2''	76° 33' 35,1''	1696
		Mata Redondo	2° 43' 15,9''	76° 36' 12,8''	1704

	Popayán	La Tetilla	2º 32' 21,7"	76º 40' 13,0"	1818
		La Claridad	2° 27' 21,9"	76° 34' 11,6"	1889
		La Claridad	2° 27´12,1"	76° 34' 06,1"	1920
		La Claridad	2° 27' 14,9"	76° 34' 06,9"	1924
		La Claridad	2° 27' 18,6"	76° 34' 09,0"	1905
		La Claridad	2° 27' 19,3"	76° 34' 12,5"	1900
		La Claridad	2° 27' 27,9"	76° 34' 08,1"	1873
		La Claridad	2° 27' 32,3"	76° 34' 01,7"	1868
		La Claridad	2° 27' 46,3"	76° 34' 04,8"	1881
	Timbío	El Naranjal	2º 18' 57,8"	76º 42' 01,2"	1848
		Las Piedras	2º 23' 33,9"	76º 42' 42,6"	1791
		Hato viejo	2º 20' 27,8"	76º 40' 13,9"	1845
		El Altillo	2º 20' 16,4"	76º 40' 42,2"	1823
		Samboní alto	2° 22' 22,3"	76° 44' 34,3"	1802
		Samboní alto	2° 22' 24,5"	76° 44' 47,4"	1808
		Samboní alto	2° 22' 31,5"	76° 44' 42,8"	1788
		Urubamba	2° 23' 34,5"	76° 43' 38,3"	1816
		Urubamba	2° 23' 40,8"	76° 43' 47,2"	1796
		Urubamba	2° 23' 41,5"	76° 43' 35,7"	1801
**Macizo**	**Sotará**	La Paz	2º 15' 35,8"	76º 40' 00,7"	2042
		La Paz	2º 15' 57,4"	76º 40' 15,2"	2137
		El Crucero	2º 22' 39,7"	76º 36' 44,8"	1940
		El Crucero	2° 22' 55,0"	76° 37' 24,3"	1944
		El Crucero	2° 22' 31,6"	76° 36' 37,8"	1987
		El Crucero	2° 22' 26,5"	76° 36' 38,1"	2020
		El Crucero	2° 22' 35,1"	76° 36' 25,5"	2008
		El Crucero	2° 22' 37,7"	76° 36' 54,5"	1985
		El Crucero	2° 22' 19,5"	76° 36' 31,4"	2016
		El Crucero	2° 22' 32,8"	76° 36' 43,6"	1993
		El Crucero	2° 22' 40,3"	76° 37' 02,5"	1941
		El Crucero	2° 22' 52,6"	76° 36' 46,8"	2027

* meters above sea level
