# Supplementary material for: Molecular Identification and Fungal Diversity Associated with Diseases in Hass Avocado Fruit Grown in Cauca, Colombia
Source: Pathogens. 2023 Dec 4;12(12):1418. doi: 10.3390/pathogens12121418 (PMC10745791; doi:10.3390/pathogens12121418)
Supplement: Supplementary file 1 [file pathogens-12-01418-s001.zip › Table S2.pdf]

Fungi associated with diseases in Hass avocado fruit			Blast isolate					
OTU	Isolates	Genbank	Genbank comparison	evalue	bit score	% query coverage	% identity	Identification
1	1H3CH	OR510139	EU851936.1	0	946	100	100	*Pseudocercospora norchiensis*
	7H3	OR510162	EU851936.1	0	946	100	100	*Pseudocercospora norchiensis*
	8H3	OR510165	EU851936.1	0	946	100	100	*Pseudocercospora norchiensis*
	13H3	OR510181	EU851936.1	0	946	100	100	*Pseudocercospora norchiensis*
	14H3CH	OR510185	EU851936.1	0	946	100	100	*Pseudocercospora norchiensis*
	15H3	OR510192	EU851936.1	0	946	100	100	*Pseudocercospora norchiensis*
	19H3CH	OR510210	EU851936.1	0	946	100	100	*Pseudocercospora norchiensis*
	20H3CH	OR510216	EU851936.1	0	946	100	100	*Pseudocercospora norchiensis*
	21H3CH	OR510221	EU851936.1	0	946	100	100	*Pseudocercospora norchiensis*
	22H3CH	OR510226	EU851936.1	0	946	100	100	*Pseudocercospora norchiensis*
	27H3CH	OR510250	EU851936.1	0	946	100	100	*Pseudocercospora norchiensis*
	31H3CH	OR510260	EU851936.1	0	946	100	100	*Pseudocercospora norchiensis*
	39H3	OR510282	EU851936.1	0	946	100	100	*Pseudocercospora norchiensis*
	40H3H	OR510289	EU851936.1	0	946	100	100	*Pseudocercospora norchiensis*
	50H3	OR510310	EU851936.1	0	946	100	100	*Pseudocercospora norchiensis*
	50H3CH	OR510311	EU851936.1	0	946	100	100	*Pseudocercospora norchiensis*
	51H3	OR510314	EU851936.1	0	946	100	100	*Pseudocercospora norchiensis*
	51H3CH	OR510315	EU851936.1	0	946	100	100	*Pseudocercospora norchiensis*
	52H3	OR510317	EU851936.1	0	946	100	100	*Pseudocercospora norchiensis*
	52H3CH	OR510318	EU851936.1	0	946	100	100	*Pseudocercospora norchiensis*
	53H3	OR510320	EU851936.1	0	946	100	100	*Pseudocercospora norchiensis*

	54H3CH	OR510322	EU851936.1	0	946	100	100	*Pseudocercospora norchiensis*
	55H3	OR510326	EU851936.1	0	946	100	100	*Pseudocercospora norchiensis*
	55H3CH	OR510327	EU851936.1	0	946	100	100	*Pseudocercospora norchiensis*
	56H3	OR510331	EU851936.1	0	946	100	100	*Pseudocercospora norchiensis*
	56H3CH	OR510332	EU851936.1	0	946	100	100	*Pseudocercospora norchiensis*
	57H3	OR510337	EU851936.1	0	946	100	100	*Pseudocercospora norchiensis*
	57H3CH	OR510338	EU851936.1	0	946	100	100	*Pseudocercospora norchiensis*
	58H3	OR510341	EU851936.1	0	946	100	100	*Pseudocercospora norchiensis*
	58H3H	OR510343	EU851936.1	0	946	100	100	*Pseudocercospora norchiensis*
	59H3CH	OR510346	EU851936.1	0	946	100	100	*Pseudocercospora norchiensis*
	62H3	OR510356	EU851936.1	0	946	100	100	*Pseudocercospora norchiensis*
	65H3	OR510359	EU851936.1	0	946	100	100	*Pseudocercospora norchiensis*
	66H3	OR510362	EU851936.1	0	946	100	100	*Pseudocercospora norchiensis*
	67H3	OR510365	EU851936.1	0	946	100	100	*Pseudocercospora norchiensis*
	67H3H	OR510367	EU851936.1	0	946	100	100	*Pseudocercospora norchiensis*
	70H3L	OR510371	EU851936.1	0	946	100	100	*Pseudocercospora norchiensis*
2	11H1	OR510172	MN723897.1	0	966	100	100	*Neopestalotiopsis* sp.
	14H1	OR510183	MN723897.1	0	966	100	100	*Neopestalotiopsis* sp.
	15H1	OR510189	MN723897.1	0	966	100	100	*Neopestalotiopsis* sp.
	15H1CH	OR510190	MN723897.1	0	966	100	100	*Neopestalotiopsis* sp.
	22H1	OR510223	MN723897.1	0	966	100	100	*Neopestalotiopsis* sp.
	22H1CH	OR510224	MN723897.1	0	966	100	100	*Neopestalotiopsis* sp.
	22H2	OR510225	MN723897.1	0	966	100	100	*Neopestalotiopsis* sp.
	24H1CH	OR510235	MN723897.1	0	966	100	100	*Neopestalotiopsis* sp.

	28H1	OR510252	MN723897.1	0	966	100	100	*Neopestalotiopsis* sp.
	29H1	OR510254	MN723897.1	0	966	100	100	*Neopestalotiopsis* sp.
	2H1	OR510142	MN723897.1	0	966	100	100	*Neopestalotiopsis* sp.
	30H1CH	OR510256	MN723897.1	0	966	100	100	*Neopestalotiopsis* sp.
	32H1	OR510263	MN723897.1	0	966	100	100	*Neopestalotiopsis* sp.
	32H1CH	OR510264	MN723897.1	0	966	100	100	*Neopestalotiopsis* sp.
	34H1CH	OR510269	MN723897.1	0	966	100	100	*Neopestalotiopsis* sp.
	36H1	OR510274	MN723897.1	0	966	100	100	*Neopestalotiopsis* sp.
	37H1	OR510277	MN723897.1	0	966	100	100	*Neopestalotiopsis* sp.
	3H1	OR510146	MN723897.1	0	966	100	100	*Neopestalotiopsis* sp.
	40H1	OR510284	MN723897.1	0	966	100	100	*Neopestalotiopsis* sp.
	44H1CH	OR510296	MN723897.1	0	966	100	100	*Neopestalotiopsis* sp.
	52H1	OR510316	MN723897.1	0	966	100	100	*Neopestalotiopsis* sp.
	57H1	OR510336	MN723897.1	0	966	100	100	*Neopestalotiopsis* sp.
	61H1CH	OR510351	MN723897.1	0	966	100	100	*Neopestalotiopsis* sp.
	7H4	OR510163	MN723897.1	0	966	100	100	*Neopestalotiopsis* sp.
	13H6	OR510182	MK110648.1	0	990	100	99,81	*Fusarium* cf. *solani*
	17H4	OR510202	MK110648.1	0	990	100	99,81	*Fusarium* cf. *solani*
	18H2CH	OR510205	MK110648.1	0	990	100	99,81	*Fusarium* cf. *solani*
	19H2CH	OR510209	MK110648.1	0	990	100	99,81	*Fusarium* cf. *solani*
3	1H1	OR510137	MK110648.1	0	990	100	99,81	*Fusarium* cf. *solani*
	23H4_1	OR510232	MK110648.1	0	990	100	99,81	*Fusarium* cf. *solani*
	39H9	OR510283	MK110648.1	0	990	100	99,81	*Fusarium* cf. *solani*
	3H2	OR510147	MK110648.1	0	990	100	99,81	*Fusarium* cf. *solani*
	40H1PEU	OR510286	MK110648.1	0	990	100	99,81	*Fusarium* cf. *solani*

	44H4	OR510297	MK110648.1	0	990	100	99,81	*Fusarium* cf. *solani*
	46H9	OR510302	MK110648.1	0	990	100	99,81	*Fusarium* cf. *solani*
	47H1	OR510303	MK110648.1	0	990	100	99,81	*Fusarium* cf. *solani*
	49H9	OR510309	MK110648.1	0	990	100	99,81	*Fusarium* cf. *solani*
	55H4	OR510328	MK110648.1	0	990	100	99,81	*Fusarium* cf. *solani*
	59H4_1	OR510348	MK110648.1	0	990	100	99,81	*Fusarium* cf. *solani*
	6H1	OR510157	MK110648.1	0	990	100	99,81	*Fusarium* cf. *solani*
	8H4CH	OR510167	MK110648.1	0	990	100	99,81	*Fusarium* cf. *solani*
4	10H2	OR510169	GU256766.1	0	1002	100	100	*Clonostachys rosea*
	16H2	OR510196	GU256766.1	0	1002	100	100	*Clonostachys rosea*
	17H16	OR510204	GU256766.1	0	1002	100	100	*Clonostachys rosea*
	17H2	OR510200	GU256766.1	0	1002	100	100	*Clonostachys rosea*
	17H3CH	OR510201	GU256766.1	0	1002	100	100	*Clonostachys rosea*
	18H13	OR510207	GU256766.1	0	1002	100	100	*Clonostachys rosea*
	20H2	OR510215	GU256766.1	0	1002	100	100	*Clonostachys rosea*
	21H13CH	OR510222	GU256766.1	0	1002	100	100	*Clonostachys rosea*
	21H2	OR510220	GU256766.1	0	1002	100	100	*Clonostachys rosea*
	26H3	OR510244	GU256766.1	0	1002	100	100	*Clonostachys rosea*
	37H13	OR510279	GU256766.1	0	1002	100	100	*Clonostachys rosea*
	46H1	OR510301	GU256766.1	0	1002	100	100	*Clonostachys rosea*
	4H2	OR510150	GU256766.1	0	1002	100	100	*Clonostachys rosea*
	61H13	OR510355	GU256766.1	0	1002	100	100	*Clonostachys rosea*
	61H4CH	OR510354	GU256766.1	0	1002	100	100	*Clonostachys rosea*
	6H2	OR510158	GU256766.1	0	1002	100	100	*Clonostachys rosea*
5	14H5CH	OR510188	MK530069.1	0	946	100	99,81	*Lasiodiplodia* sp.

	17H5	OR510203	MK530069.1	0	946	100	99,81	*Lasiodiplodia* sp.
	1H5CH	OR510141	MK530069.1	0	946	100	99,81	*Lasiodiplodia* sp.
	22H5CH	OR510228	MK530069.1	0	946	100	99,81	*Lasiodiplodia* sp.
	30H5	OR510257	MK530069.1	0	946	100	99,81	*Lasiodiplodia* sp.
	34H5	OR510271	MK530069.1	0	946	100	99,81	*Lasiodiplodia* sp.
	34H5CH	OR510272	MK530069.1	0	946	100	99,81	*Lasiodiplodia* sp.
	36H5	OR510275	MK530069.1	0	946	100	99,81	*Lasiodiplodia* sp.
	3H5	OR510148	MK530069.1	0	946	100	99,81	*Lasiodiplodia* sp.
	40H5	OR510292	MK530069.1	0	946	100	99,81	*Lasiodiplodia* sp.
	4H5CH	OR510151	MK530069.1	0	946	100	99,81	*Lasiodiplodia* sp.
	68H5CH	OR510370	MK530069.1	0	946	100	99,81	*Lasiodiplodia* sp.
	6H5	OR510159	MK530069.1	0	946	100	99,81	*Lasiodiplodia* sp.
	6H5CH	OR510160	MK530069.1	0	946	100	99,81	*Lasiodiplodia* sp.
6	11H5	OR510174	OQ672358.1	0	1020	100	100	*Neofusicoccum parvum*
	24H5	OR510238	OQ672358.1	0	1020	100	100	*Neofusicoccum parvum*
	26H5	OR510245	OQ672358.1	0	1020	100	100	*Neofusicoccum parvum*
	26H5CH	OR510246	OQ672358.1	0	1020	100	100	*Neofusicoccum parvum*
	35H5CH	OR510273	OQ672358.1	0	1020	100	100	*Neofusicoccum parvum*
	45H5CH	OR510300	OQ672358.1	0	1020	100	100	*Neofusicoccum parvum*
	50H5	OR510312	OQ672358.1	0	1020	100	100	*Neofusicoccum parvum*
	58H5CH	OR510344	OQ672358.1	0	1020	100	100	*Neofusicoccum parvum*
	5H5	OR510155	OQ672358.1	0	1020	100	100	*Neofusicoccum parvum*
	65H5	OR510361	OQ672358.1	0	1020	100	100	*Neofusicoccum parvum*
	70H5CHP	OR510373	OQ672358.1	0	1020	100	100	*Neofusicoccum parvum*
7	12H5	OR510178	MT587514.1	0	1022	100	100	*Neofusicoccum ribis*

	19H5	OR510212	MT587514.1	0	1022	100	100	*Neofusicoccum ribis*
	23H5CH	OR510234	MT587514.1	0	1022	100	100	*Neofusicoccum ribis*
	2H5CH	OR510145	MT587514.1	0	1022	100	100	*Neofusicoccum ribis*
	40H5Eu	OR510294	MT587514.1	0	1022	100	100	*Neofusicoccum ribis*
	60H5CH	OR510350	MT587514.1	0	1022	100	100	*Neofusicoccum ribis*
	70H5CHL	OR510372	MT587514.1	0	1022	100	100	*Neofusicoccum ribis*
8	15H4CH	OR510194	MN856281.1	0	1011	100	100	*Colletotrichum kahawae*
	25H4	OR510241	MN856281.1	0	1011	100	100	*Colletotrichum kahawae*
	2H4	OR510144	MN856281.1	0	1011	100	100	*Colletotrichum kahawae*
	32H4	OR510266	MN856281.1	0	1011	100	100	*Colletotrichum kahawae*
	45H4	OR510299	MN856281.1	0	1011	100	100	*Colletotrichum kahawae*
	49H4CH	OR510307	MN856281.1	0	1011	100	100	*Colletotrichum kahawae*
	5H4	OR510153	MN856281.1	0	1011	100	100	*Colletotrichum kahawae*
9	12H2CH	OR510177	MF070233.1	0	1014	100	99,82	*Diaporthe* sp.
	15H2	OR510191	MF070233.1	0	1014	100	99,82	*Diaporthe* sp.
	17H1	OR510199	MF070233.1	0	1014	100	99,82	*Diaporthe* sp.
	29H2	OR510255	MF070233.1	0	1014	100	99,82	*Diaporthe* sp.
	34H1	OR510268	MF070233.1	0	1014	100	99,82	*Diaporthe* sp.
	56H1CH	OR510330	MF070233.1	0	1014	100	99,82	*Diaporthe* sp.
10	10H4	OR510170	MN565960.1	0	1011	100	100	*Colletotrichum gloeosporioides* species complex
	15H4	OR510193	MN565960.1	0	1011	100	100	*Colletotrichum gloeosporioides* species complex
	20H4	OR510217	MN565960.1	0	1011	100	100	*Colletotrichum gloeosporioides* species complex
	34H4	OR510270	MN565960.1	0	1011	100	100	*Colletotrichum gloeosporioides* species complex

	59H4	OR510347	MN565960.1	0	1011	100	100	*Colletotrichum gloeosporioides* species complex
	62H4	OR510357	MN565960.1	0	1011	100	100	*Colletotrichum gloeosporioides* species complex
11	11H2	OR510173	MT150623.1	0	1011	99	99,82	*Diaporthe* sp.
	12H2	OR510176	MT150623.1	0	1011	99	99,82	*Diaporthe* sp.
	40H2	OR510287	MT150623.1	0	1011	99	99,82	*Diaporthe* sp.
	55H2	OR510325	MT150623.1	0	1011	99	99,82	*Diaporthe* sp.
	5H2	OR510152	MT150623.1	0	1011	99	99,82	*Diaporthe* sp.
12	20H5CH	OR510219	MT028605.1	0	1020	100	100	*Diaporthe* sp.
	36H5CH	OR510276	MT028605.1	0	1020	100	100	*Neofusicoccum dianense*
	40H5Pe	OR510295	MT028605.1	0	1020	100	100	*Neofusicoccum dianense*
	57H5CH	OR510339	MT028605.1	0	1020	100	100	*Neofusicoccum dianense*
13	58H3CH	OR510342	MG976377.1	0	1007	99	99,82	*Diaporthe* sp.
	61H2CH	OR510352	MG976377.1	0	1007	99	99,82	*Diaporthe* sp.
	7H2	OR510161	MG976377.1	0	1007	99	99,82	*Diaporthe* sp.
	8H2	OR510164	MG976377.1	0	1007	99	99,82	*Diaporthe* sp.
14	18H4	OR510206	MK426765.1	0	1013	100	100	*Colletotrichum gloeosporioides* species complex
	23H4CH	OR510233	MK426765.1	0	1013	100	100	*Colletotrichum gloeosporioides* species complex
	33H4	OR510267	MK426765.1	0	1013	100	100	*Colletotrichum gloeosporioides* species complex
	56H4	OR510333	MK426765.1	0	1013	100	100	*Colletotrichum gloeosporioides* species complex
15	14H4	OR510186	MT549849.1	0	981	100	100	*Fusarium fujikuroi* species complex
	19H4CH	OR510211	MT549849.1	0	981	100	100	*Fusarium fujikuroi* species complex
	24H4CH	OR510237	MT549849.1	0	981	100	100	*Fusarium fujikuroi* species complex
	65H4	OR510360	MT549849.1	0	981	100	100	*Fusarium fujikuroi* species complex

16	40H5CH	OR510293	MT332314.1	0	952	100	100	*Lasiodiplodia pseudotheobromae*
	49H5	OR510308	MT332314.1	0	952	100	100	*Lasiodiplodia pseudotheobromae*
	50H5CH	OR510313	MT332314.1	0	952	100	100	*Lasiodiplodia pseudotheobromae*
	54H5CH	OR510324	MT332314.1	0	952	100	100	*Lasiodiplodia pseudotheobromae*
17	31H9	OR510261	OP897165.1	0	998	99	99,1	*Diaporthe* sp.
	31H9CH	OR510262	OP897165.1	0	998	99	99,1	*Diaporthe* sp.
	38H9CH	OR510281	OP897165.1	0	998	99	99,1	*Diaporthe* sp.
18	27H2	OR510249	MT611201.1	0	1007	100	99,64	*Diaporthe* sp.
	28H2	OR510253	MT611201.1	0	1007	100	99,64	*Diaporthe* sp.
	47H2	OR510304	MT611201.1	0	1007	100	99,64	*Diaporthe* sp.
19	23H4	OR510231	MH865609.1	0	1009	99	100	*Colletotrichum fructicola*
	37H4	OR510278	MH865609.1	0	1009	99	100	*Colletotrichum fructicola*
	8H4	OR510166	MH865609.1	0	1009	99	100	*Colletotrichum fructicola*
20	40H4	OR510290	MT611204.1	0	1013	100	100	*Colletotrichum gloeosporioides* species complex
	55H4CH	OR510329	MT611204.1	0	1013	100	100	*Colletotrichum gloeosporioides* species complex
	67H4CH	OR510368	MT611204.1	0	1013	100	100	*Colletotrichum gloeosporioides* species complex
21	19H2	OR510208	MW546045.1	0	996	99	99,82	*Diaporthe* sp.
	31H2	OR510259	MW546045.1	0	996	99	99,82	*Diaporthe* sp.
	32H2	OR510265	MW546045.1	0	996	99	99,82	*Diaporthe* sp.
22	19H15	OR510213	MT598163.1	0	957	100	100	*Fusarium* cf. *graminearum*
	26H15	OR510247	MT598163.1	0	957	100	100	*Fusarium* cf. *graminearum*
	3H15	OR510149	MT598163.1	0	957	100	100	*Fusarium* cf. *graminearum*
23	27H1CH	OR510248	MT163275.1	0	1070	100	100	*Pestalotiopsis* sp.
	59H1CH	OR510345	MT163275.1	0	1070	100	100	*Pestalotiopsis* sp.
24	20H1	OR510214	KU504326.1	0	1035	100	100	*Sarocladium* sp.

	67H3CH	OR510366	KU504326.1	0	1035	100	100	*Sarocladium* sp.
25	16H4	OR510197	KJ627843.1	0	1024	100	99,82	*Colletotrichum acutatum* species complex
	25H4_1	OR510242	KJ627843.1	0	1024	100	99,82	*Colletotrichum acutatum* species complex
26	16H4CH	OR510198	MH893689.1	0	1029	100	100	*Colletotrichum acutatum* species complex
	54H4	OR510323	MH893689.1	0	1029	100	100	*Colletotrichum acutatum* species complex
27	40H4CH	OR510291	KX673575.1	0	1024	100	99,82	*Colletotrichum acutatum* species complex
	56H4CH	OR510334	KX673575.1	0	1024	100	99,82	*Colletotrichum acutatum* species complex
28	56H5	OR510335	OQ672358.1	0	1014	100	99,82	*Neofusicoccum parvum*
	5H5Pic	OR510156	OQ672358.1	0	1014	100	99,82	*Neofusicoccum parvum*
29	23H2CH	OR510230	EU002927.1	0	1013	99	100	*Diaporthe* sp.
	26H2CH	OR510243	EU002927.1	0	1013	99	100	*Diaporthe* sp.
30	52H4CH	OR510319	MT470601.1	0	1013	99	100	*Colletotrichum theobromicola*
	66H4	OR510363	MT470601.1	0	1013	99	100	*Colletotrichum theobromicola*
31	2H2	OR510143	MH236147.1	0	959	100	98,19	*Diaporthe* sp.
	40H1EU	OR510285	MH236147.1	0	959	100	98,19	*Diaporthe* sp.
32	20H4CH	OR510218	JN715847.1	0	1011	100	100	*Colletotrichum kahawae*
	22H4CH	OR510227	JN715847.1	0	1011	100	100	*Colletotrichum kahawae*
33	58H2	OR510340	KX385048.1	0	998	100	100	*Fusarium solani* species complex
	64H2	OR510358	KX385048.1	0	998	100	100	*Fusarium solani* species complex
34	16H1CH	OR510195	OQ756171.1	0	987	99	99,81	*Fusarium* cf. *solani*
	38H9	OR510280	OQ756171.1	0	987	99	99,81	*Fusarium* cf. *solani*
35	10H5	OR510171	OK268021.1	0	950	99	100	*Lasiodiplodia* sp.
	27H5CH	OR510251	OK268021.1	0	950	99	100	*Lasiodiplodia* sp.
36	1H2	OR510138	MT992059.1	0	942	96	98,87	*Diaporthe* sp.
37	1H4CH	OR510140	KX344998.1	0	1029	100	100	*Colletotrichum acutatum* species complex
38	5H4TO	OR510154	MH865701.1	0	1026	99	100	*Colletotrichum tamarilloi*
39	8H12	OR510168	MH863698.1	0	981	99	100	*Colletotrichum gigasporum*

40	12H1	OR510175	MT560227.1	0	959	100	100	*Fusarium incarnatum-equiseti* species complex
41	12H9	OR510179	OP897224.1	0	1000	99	99,1	*Diaporthe* sp.
42	13H2	OR510180	EU715618.1	0	972	98	98,72	*Diaporthe* sp.
43	14H2	OR510184	MG976413.1	0	1007	99	99,82	*Diaporthe* sp.
44	14H5	OR510187	MK217323.1	0	1020	100	100	*Neofusicoccum kwambonambiense*
45	22H10	OR510229	OR225859.1	0	939	99	99,23	*Fusarium* cf. *solani*
46	24H2CH	OR510236	ON329677.1	0	1122	100	99,84	*Schizophyllum commune*
47	25H2	OR510239	OP897178.1	0	1007	99	99,82	*Diaporthe* sp.
48	25H2CH	OR510240	MT601890.1	0	1014	100	99,82	*Diaporthe* sp.
49	31H1	OR510258	OQ818136.1	0	998	100	100	*Fusarium solani* species complex
50	40H3	OR510288	EU851911.1	0	946	100	100	*Pseudocercospora norchiensis*
51	45H1	OR510298	MH003453.1	0	1013	100	99,64	*Diaporthe* sp.
52	47N	OR510305	JF773672.1	0	994	99	99,63	*Diaporthe* sp.
53	49H4	OR510306	MT597824.1	0	1011	100	100	*Colletotrichum gloeosporioides* species complex
54	53H4	OR510321	MT065696.1	0	1013	100	100	*Colletotrichum gloeosporioides*
55	59H4CH	OR510349	MN013859.1	0	968	100	99,07	*Fusarium solani* species complex
56	61H3	OR510353	MT597828.1	0	972	100	100	*Penicillium citrinum*
57	66H5CH	OR510364	MN634051.1	0	1020	100	100	*Neofusicoccum parvum*
58	67H9	OR510369	OP897224.1	0	998	99	99,1	*Diaporthe* sp.
